# Conformational diversity analysis reveals three functional mechanisms in proteins

**DOI:** 10.1371/journal.pcbi.1005398

**Published:** 2017-02-13

**Authors:** Alexander Miguel Monzon, Diego Javier Zea, María Silvina Fornasari, Tadeo E. Saldaño, Sebastian Fernandez-Alberti, Silvio C. E. Tosatto, Gustavo Parisi

**Affiliations:** 1 Departamento de Ciencia y Tecnología, Universidad Nacional de Quilmes (CONICET), Bernal, Buenos Aires, Argentina; 2 Bioinformatics Unit, Fundación Instituto Leloir (CONICET), Buenos Aires, Argentina; 3 Department of Biomedical Sciences, University of Padua, Padua, Italy; University College London, UNITED KINGDOM

## Abstract

Protein motions are a key feature to understand biological function. Recently, a large-scale analysis of protein conformational diversity showed a positively skewed distribution with a peak at 0.5 Å C-alpha root-mean-square-deviation (RMSD). To understand this distribution in terms of structure-function relationships, we studied a well curated and large dataset of ~5,000 proteins with experimentally determined conformational diversity. We searched for global behaviour patterns studying how structure-based features change among the available conformer population for each protein. This procedure allowed us to describe the RMSD distribution in terms of three main protein classes sharing given properties. The largest of these protein subsets (~60%), which we call “rigid” (average RMSD = 0.83 Å), has no disordered regions, shows low conformational diversity, the largest tunnels and smaller and buried cavities. The two additional subsets contain disordered regions, but with differential sequence composition and behaviour. Partially disordered proteins have on average 67% of their conformers with disordered regions, average RMSD = 1.1 Å, the highest number of hinges and the longest disordered regions. In contrast, malleable proteins have on average only 25% of disordered conformers and average RMSD = 1.3 Å, flexible cavities affected in size by the presence of disordered regions and show the highest diversity of cognate ligands. Proteins in each set are mostly non-homologous to each other, share no given fold class, nor functional similarity but do share features derived from their conformer population. These shared features could represent conformational mechanisms related with biological functions.

## Author summary

Protein motions are commonly quantified measuring structural differences between conformers. The extension of these differences are called conformational diversity. These motions are essential to understand protein biology. We have found that the distribution of conformational diversity in a large dataset of proteins could be explained in terms of three sets sharing structure-based features emerging from the conformer population for each protein. The first set, which we called rigid, involve proteins showing almost no backbone movements but with important changes in tunnels. In order of increasing conformational diversity, the other sets are called partially disordered and malleable, showing disordered regions and important cavities but with different behaviour to each other. Shared features in each set could represent conformational mechanisms related with biological functions.

## Introduction

Early crystallization studies on myoglobin found no apparent way the oxygen could possibly enter the molecule and bind to heme [1]. It took more than a decade to discover that protein motions were essential for myoglobin to be biologically active [2,3]. After these early findings, an overwhelming amount of information has accumulated relating protein motion with biological function. A wide range of movements have been explored in proteins, from large relative domain movements [4], secondary and tertiary element rearrangements [5] and loop displacements [6] to small residue rearrangements [7]. The upper limit in this scale of protein movements may certainly involve intrinsically disordered regions (IDRs) or proteins (IDPs) characterized by their high flexibility and mobility and clearly related with well-established disorder-based biological functions [8] although other notion of disorder role has been proposed [9].

A large-scale survey of protein motion degrees, studying the extension of protein conformational diversity using a redundant collection of crystallized structures for the same protein was recently published [10]. Since the early determination of haemoglobin conformers, it is generally accepted that different crystallographic structures for the same protein (i.e. with and without substrate or post-translational modifications) could represent putative instances of the conformational space of a protein [11]. To measure the structural differences between putative conformers, Burra and co-workers used C-alpha root mean square deviation (RMSD). Clearly, RMSD or other structural similarity scores measure the differences in ordered parts of the proteins and stress the importance of protein motions in the known protein structure space. From this distribution, it is possible to infer how a great majority of proteins have RMSD values compatible with the accepted error in estimating a structure using X-ray crystallography (about 0.4 Å). The obtained distribution is consistent with other works reporting low degrees of conformational diversity in proteins. In a study of conformational changes in 60 enzymes between their apo and substrate-bound forms, 75% of the data had an RMSD less than 1 Å, and 91% less than 2 Å, with an average of 0.7 Å [12]. Interestingly, comparisons of apo structures of the same protein show an RMSD of 0.5 Å, a value slightly below the observed apo and substrate-bound average. In agreement with these results, large-scale protein motions are not necessary to sustain biological function in the majority of proteins studied. This observation is supported with the finding that even small changes between conformers could greatly affect catalytic parameters and biological behaviour of enzymes [13,14]. Also, it has been suggested that key and widely extended biological properties in proteins [15], such as allosterism and cooperativism, could arise from changes in the width of conformational distributions without any appreciable change in the average structure of the protein [16]. However, the distribution of Burra et al. shows also a large skew towards higher RMSD (observed maximum RMSD = 23.7 Å) indicating that a minor fraction of the analysed dataset does require large conformational changes to be functionally active [10]. High RMSD values, are commonly observed in multidomain proteins where hinge motions produce relative movements of domains as rigid bodies [17].

As pointed out by Fraunfelder, the search for general concepts between dynamics and protein function is a key issue in structural biology [2]. Knowing the degree of conformational changes could contribute highly to our understanding of how these motions modulate protein function. Large-scale analysis of different degrees and types of protein motions could also allow us to infer general rules about different structure-function relationships in protein structural space. In this work, we have used a large and well curated set of proteins derived from the CoDNaS (Conformational Diversity of Native State) database [18] to reproduce Burra et al distribution. Using several structural and dynamical analyses over the available population of conformers for each protein, we found that three main structure-function relationships emerge. Proteins in each group are mostly non-homologous and do not show any fold preference or functional similarity. However, proteins in each set share similar features emerged from conformational analysis, which as a global behaviour could represent conformational mechanisms related with protein function.

## Results

### General distribution and modulating factors

CoDNaS [18] is a database which contains a redundant collection of three-dimensional structures for the same sequence where each structure could be taken as snapshot of the conformational ensemble of the protein [11]. Essentially, for each protein in CoDNaS, we performed an extensive pairwise structural comparison between all conformers and derived different structural similarity scores linked with the corresponding information of the structure determination. In this way, it is easy to study structural differences between conformers and relate them to a given chemical and/or biological property characterizing conformers.

**Fig 1:**
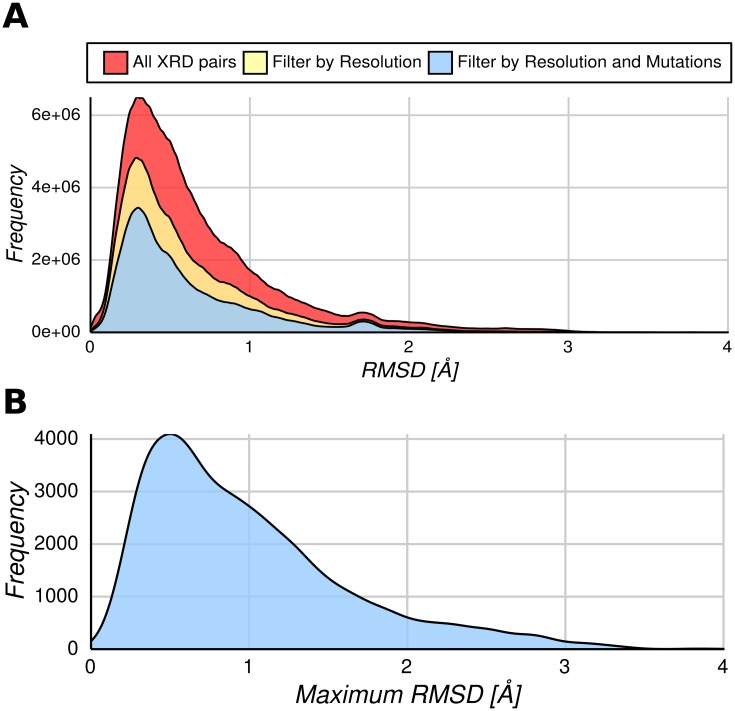
Global conformational diversity distributions of proteins in CoDNaS. (A) All pairwise RMSDs values between proteins with conformers obtained by X-ray diffraction (XRD) (light red distribution). The light yellow distribution considered only pairs of conformers which were obtained at resolution less than 2.5 Å and the light red distribution just conformers without mutations. (B) Maximum conformational diversity distribution of pairs of conformers with the maximum RMSD value per protein used in the rest of the manuscript.

In Fig 1A, we show the general distribution of RMSD between conformers for all protein chains contained in CoDNaS obtained by X-Ray crystallography (~16,000 different protein chains). However, the presence of mutations, low resolution structures and the number of structures could affect the assessment of the degree of conformational diversity [19]. Additionally, as less than 6% of CoDNaS entries have NMR estimated structures and due to their differential flexibility behaviour compared with X-ray crystallographic structures [20], we removed conformers for each protein determined by this method in order to avoid bias produced by mixing NMR and X-Ray structures. In this sense, we used a reduced dataset (4,791 different protein chains with 74,417 conformers and 1,186,312 pairs of conformers) in order to avoid conformational diversity biases (see Methods). All distributions in Fig 1A show the previously identified trend [10]. In our analysis, it is also possible to study the maximum conformational diversity shown by a given chain or protein (the pair of conformers showing the maximum RMSD between all pair-wise comparisons for a given protein). This distribution (Fig 1B) again follows the general trend, with a median of 0.83 Å, an average of 0.99 Å and a large peak near 0.4 Å. It is possible to infer from this figure that most proteins require small movements between conformers to accomplish their biological functions.

### Mining the distribution

**Fig 2:**
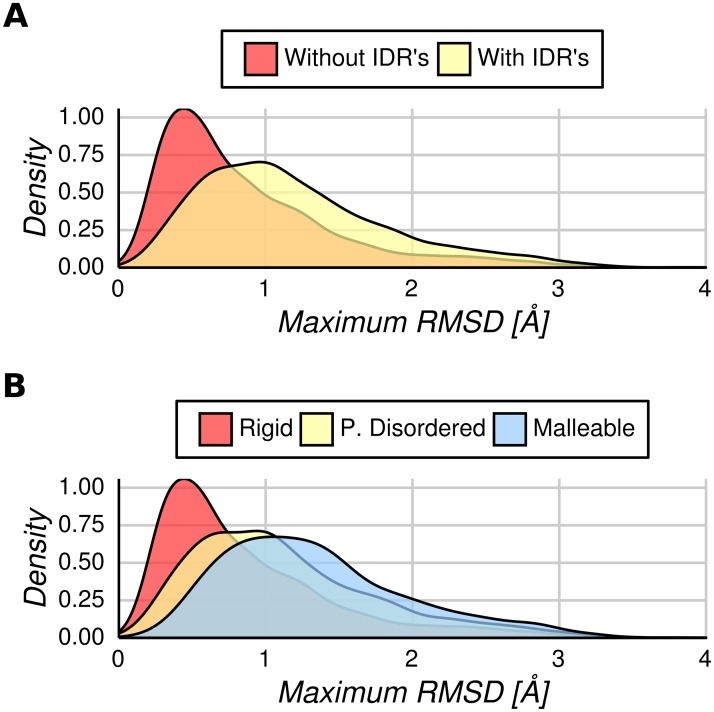
Maximum conformational diversity distributions. (A) Pairs of conformers showing maximum RMSD for proteins with/without IDRs considering all the available conformers per protein. In light red, we show the distribution without IDRs and in light yellow proteins with IDRs in at least one of their conformers. The distribution of proteins without IDRs had significantly lower overall RMSD values compared with proteins with IDRs (Kolmogorov—Smirnov test, P << 0.001). (B) The maximum conformational diversity distribution can be represented by three main sets of proteins: rigid (all conformers per protein without IDRs), partially disordered (with IDRs at least in one conformer and in the maximum RMSD pair of conformational diversity) and malleable (with IDRs in at least one conformer but the maximum pair of conformational diversity without IDRs). These three distributions are significantly different in their median values (Kruskal—Wallis rank sum test, P << 0.001 with a Nemenyi post-hoc test).

We have recently found that proteins showing order-disorder transitions between conformers show higher RMSD values than proteins showing no transitions [21]. In that study, the pair of *apo* and *holo* forms with the largest transition was selected and the RMSD was estimated using their common folded regions. This finding induced us to explore the role of disordered regions in the conformational diversity of folded regions in proteins. Using our working definition of disordered regions (see Methods) mapping IDRs on all conformers of a given protein, allowed us to split the maximum RMSD distribution (Fig 1B) into two new distributions shown in Fig 2A. One of the maximum RMSD distributions (light red in Fig 2A) with an average RMSD of 0.87 Å contains ordered proteins with no IDRs (in any of their available conformers). While the other has an average RMSD of 1.20 Å (light yellow in Fig 2A), and contains proteins with IDRs (in at least one of their available conformers). These distributions are statistically different in shape and median (Kolmogorov-Smirnov test and Wilcoxon rank-sum test respectively, P << 0.001).

As proteins with IDRs have higher maximum RMSD of alpha carbons between conformers, this value could be used to predict whether that protein is disordered (at least one conformer with IDRs) or ordered (Fig 2A). The Area Under the Receiver Operating Characteristic (ROC) Curve (AUC) for maximum RMSD as a predictor of disordered proteins is 0.67. Also, logistic regression for predicting disordered proteins using maximum RMSD values, shows a statistically significant coefficient (P < 0.001). For a cutoff of 0.9 Å maximum RMSD, the accuracy to predict presence of disorder is 64% and for RMSD > 1.2 Å the accuracy is 66%. Above 0.9 Å of maximum RMSD we found 3.03 times more proteins with disordered regions than below it. These results show that proteins with IDRs have higher RMSD values in their structured part than fully ordered proteins.

Furthermore, the presence of disordered regions could still be used to separate the distribution of proteins with IDRs. Pairs of conformers showing maximum RMSD could contain or not disordered regions. We found that proteins with IDRs in the maximum RMSD pair have an average RMSD of 1.14 Å, while those without IDRs in the maximum RMSD pair have an average RMSD of 1.35 Å (Kolmogorov-Smirnov test and Wilcoxon rank-sum test, P-value << 0.01). In increasing order of higher conformational diversity, we call these three distributions “rigid” with average RMSD = 0.85 Å (light red in Fig 2B), “partially disordered” with average RMSD = 1.1 Å (light yellow in Fig 2B) and finally “malleable” with average RMSD = 1.3 Å (light blue in Fig 2B). These three distributions are significantly different in their median values (Kruskal—Wallis rank sum test, P < 0.001 with a Nemenyi post-hoc test). We will show later that, based on their structural and dynamical features, each of these distributions could represent different global conformational mechanisms.

Importantly, maximum RMSD and the number of conformers available for each protein have a negligible Spearman’s correlation coefficient (rho = 0.094), nor with protein length [10]. Also, the percentage of disordered conformers characterizing the partially disordered and malleable sets with the number of conformers per protein have a very weak Spearman’s correlation coefficient (rho = 0.038).

### Structural characterization of the distributions

It has been suggested that temperature used in structure estimation as well as the presence of crystallographic contacts can introduce biases in protein conformation. We found that the maximum RMSD distributions of the rigid, partially disordered and malleable sets are robust to the temperature used in structure estimation (room or cryogenic temperatures) and to the mean number of crystallographic contacts (please see supplementary Figure A-C in S1 and S2 Figs).

S1 Table summarizes several structural characterizations and composition of the rigid, partially disordered and malleable protein sets. It contains the mean, median and standard deviation of the compared distributions. The rigid protein distribution has the lowest mean RMSD and no disordered regions in all their available conformers. This is the most populated of the three groups (64.18% of the analysed proteins). We found that the partially disordered and malleable sets differ in the number of conformers with IDRs. The partially disordered set have 69.1% of their conformers with at least one disordered region, while the malleable set has only 24.86% of conformers showing IDRs. This difference is also evident in the amino acid composition of these regions. Thus, we compared the amino acid composition of disordered regions in our sets with the DisProt [22] database (a database of proteins with experimentally determined disorder regions) relative to PDB Select 25 [23] (proteins amenable to crystallization studies based on ordered residues). We found that IDRs in partially disordered and malleable sets are rich in amino acids characterizing flexible regions and depleted in amino acids related with globularity or foldability. This observation can be derived from the distribution shown in S3 Fig, where amino acids proportions are ordered using the Vihinen amino acid flexibility distribution [24]. It is also possible to observe that the partially disordered and malleable sets show similar amino acid composition, but with certain differences. Malleable proteins show a higher proportion of His, Thr and Lys and are depleted in Ala when compared with the partially disordered set, which in turn show an oddly higher proportion of Pro compared with malleable proteins and DisProt.

The differences in IDRs compositions of these sets directly impact the corresponding RMSD distributions. As derived from S1 Table, partially disordered proteins have more IDRs showing a higher average percent of disorder and also longer IDRs compared to the malleable set. Malleable proteins show no IDRs in the maximum RMSD pair, mainly because their IDRs are mostly ordered in their conformers. However, these regions are highly flexible, introducing higher RMSD values when become ordered. On the contrary, highly flexible regions in partially disordered proteins are mostly disordered and do not impact the RMSD estimation. A direct consequence of this observation is observed when RMSD is estimated using only loop regions. We found that again malleable proteins show higher values than partially disordered proteins (see Figure A in S4 Fig) while no statistically significant differences were found using other secondary structures (see Figure B-C in S4 Fig). Furthermore, when removing all regions that appear disordered in at least one conformer available for each protein and recalculating the RMSD, we found no difference between partially disordered and malleable populations (Wilcoxon rank-sum test P = 0.74 and Kolmogorov-Smirnov test P = 0.83). In fact, the average RMSD of the three sets are very similar to each other with an average re-estimated RMSD of 0.86 Å, 0.993 Å and 0.995 Å for the rigid, partially disordered and malleable sets respectively. This supports the vision that conformational diversity of the three main groups mainly differs in the conformation of ordered regions involved in order/disorder transitions but with different tendencies to transit between these states.

Other structural differences were also found between the sets. For example, partially disordered proteins contain more than twice as many hinges as malleable and rigid proteins (S5 Fig) and also higher normalized radius of gyration (S6 Fig). The presence of hinges may be related with an increase in conformational diversity due to relative domain movements, which could be supported by a slightly higher average of domains for partially disordered proteins (1.61 compared to 1.50 in malleable and 1.49 in rigid). On the other hand, the increased normalized radius of gyration in partially disordered proteins could evidence larger fold volumes and less compactness than in the other sets.

Conformers in our dataset could differ in several conditions associated to the corresponding crystallization conditions (for example post-translational modifications, different oligomeric structure, presence of ligands). Although it is difficult to establish an exact correlation between structural differences among conformers and protein function, we studied how the presence of the three sets are represented using just *apo* and *holo* conformers in the maximum pair of RMSD for each protein (see the composition of this subset in S2 Table). We found (S7 Fig) that using this subset of *apo* and *holo* conformer pairs we obtained the three main populations of rigid, partially disordered and malleable again significantly different in their median and they follow the same tendency as the original dataset (Kruskal—Wallis rank sum test, P < 0.001 with a Nemenyi post-hoc test).

Finally, mapping protein domains with CATH showed that more than 84% of the folds for rigid proteins correspond to different superfamilies. The same behaviour is found in partially disordered and malleable proteins with 74% and 78%. The most populated cluster represents 2%, 6% and 3.4% of each set for rigid, partially disordered and malleable sets. Sequence clustering at 30% identity produce 57%, 72% and 80% of one-member clusters for the rigid, partially disordered and malleable sets respectively. These results are consistent with the idea that the majority of the proteins in each group are not homologous.

### Backbone-independent conformational diversity characterization

**Fig 3:**
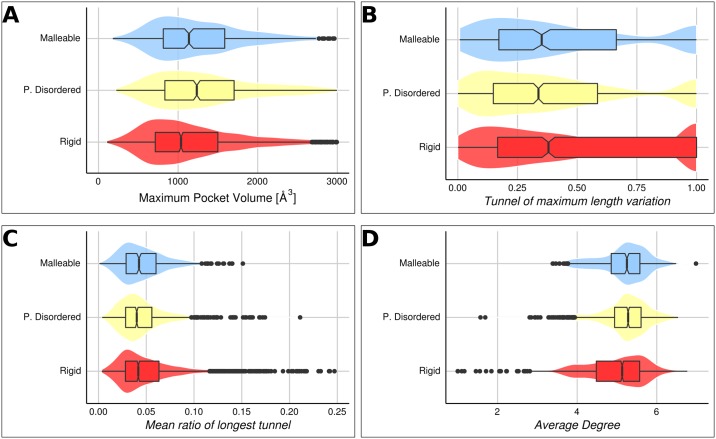
Backbone-independent conformational diversity characterization. On each boxplot, bottom and top of the box correspond to the first and third quartile, the vertical bar inside the box is the median (Second quartile) and the notches displays the median absolute deviation (M.A.D). Also, the violin plot under the boxplot shows the probability density of given variable. (A) Maximum pocket volume distribution between conformers in maximum pair of conformational diversity. Cavities are significantly greater in partially disordered and malleable proteins (Kruskal—Wallis rank sum test shows significant differences between this groups P << 0.001, with a Nemenyi post-hoc test shows that the volume of cavities in rigid proteins are significantly different of the cavity volumes in partially disordered or malleable proteins with P < 0.001). (B) Maximum tunnel length variation distribution between conformers in maximum pair of conformational diversity. The parameter expresses the proportion of variation between largest tunnels in each conformer and it is calculated as |*L*_1_ − *L*_2_ |/max(*L*_1_,*L*_2_), where L_i_ is the length of the largest tunnel on the corresponding conformer in the maximum pair. The mean value in rigid proteins is significantly greater than partially disordered proteins (Wilcoxon rank sum test, P < 0.001). (C) Mean ratio of longest tunnel distribution between conformers in maximum pair of conformational diversity. The tunnel length is normalized by the conformer length. (D) Average degree distributions between conformers in maximum pair of conformational diversity. Partially disordered proteins show greater mean values than rigid proteins (Kruskal—Wallis rank sum test shows significant differences between this groups P << 0.001 with a Nemenyi post-hoc test).

We have mentioned that large conformational changes are not required for proteins to sustain biological activity. Furthermore, low RMSD values as those found in rigid proteins (average RMSD = 0.85 Å) does not mean they lack conformational changes which may be involved in biological processes as previously indicated for allosteric proteins [25]. We study the presence of cavities and tunnels and their variation among conformers in order to study conformational diversity almost independent of carbon-alpha displacements. Cavities and tunnels are structures that connect the protein surface with buried active or binding sites in proteins and are essential for biological activity in most proteins [7,26]. Using Fpocket [27] to characterize the presence of cavities in each conformer (usually those with higher volume values), we observe how cavities are larger in partially disordered and malleable proteins (Fig 3A). Figures were represented as boxplots and violin superposed plots to facilitate evaluation and interpretation of the compared distributions (this information is also included in S1 Table). This estimation was performed using ordered pairs of conformers of the corresponding sets. While partially disordered and malleable proteins have similar volume cavities, they have important differences in their behaviour. Highly flexible regions commonly found in their ordered form in malleable proteins could define larger cavities and higher volume variation than in other sets. When IDRs in any of their conformers are removed, cavities in malleable proteins greatly reduce their volume while partially disordered cavities remain almost unaltered (see S1 Table). IDRs and highly flexible regions form and define, at least partially, the cavities of malleable proteins possibly modulating interaction with ligands which are more diverse in these proteins. Cavities in partially disordered and malleable sets are also more solvent exposed and more hydrophilic than those observed in rigid proteins in terms of pocket density and ASA values.

The size and variation of tunnels between conformers were also studied using MOLE 2.0 [28]. Considering the tunnel of maximum length and its variation among conformers, larger values are obtained in rigid proteins (Fig 3B). Opening and closing of main tunnels between conformers are unequivocally related with biological function [7]. Interestingly, we found that RMSD per position for tunnel lining residues is larger when compared with residues outside tunnels. This difference is only statistically significant when analysed rigid proteins (Kolmogorov-Smirnov test and Wilcoxon rank sum tests, P < 0.01). Furthermore, rigid proteins have statistically more important tunnels than partially disordered and malleable proteins, as measured as the ratio of the longest tunnel (normalized for protein length) in the maximum RMSD conformer pair (Kruskal—Wallis rank sum test P = 0.01 with Nemenyi post-hoc test) (Fig 3C). Interestingly, these measures are not statistically different when partially disordered and malleable proteins are compared to each other. Additionally, we found that ligand molecular weights are bigger in malleable and partially disordered sets when compared with rigid proteins (average molecular weights: 414.12, 429.27 and 388.92 respectively).

Slight conformational changes were also studied as the average degree (contacts between residues) of the residue interaction network using RING [29]. We found (Fig 3D) that rigid proteins have lower average contacts per residue than partially disordered and malleable proteins (average degree: 5, 5.21 and 5.16 respectively, Kruskal-Wallis rank sum with Nemenyi post-hoc test P << 0.001). Presence of more connected networks in the proteins with order-disorder transitions may be associated with an adaptive evolutionary process to compensate the presence of highly flexible regions in the ordered part of the protein.

## Discussion

**Fig 4:**
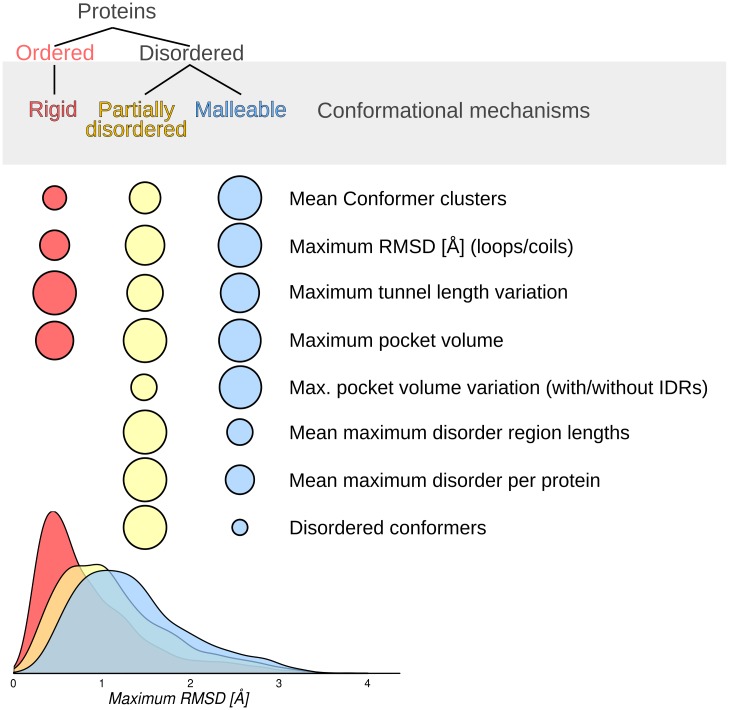
Visual comparison of main structural features in each of the three protein sets described in this work. Area of each circle is proportional to the average corresponding quantitative measure.

We have found that the distribution of protein conformational diversity can be explained in terms of three main protein sets. The differences between them do not rely on structural classification, biological activity nor common evolutionary history. The average behaviour of the proteins in each set results from shared structural and dynamical features when their conformational populations are considered. The features shared between proteins in the same set could be seen as conformational mechanisms associated with protein function (Fig 4).

**Fig 5:**
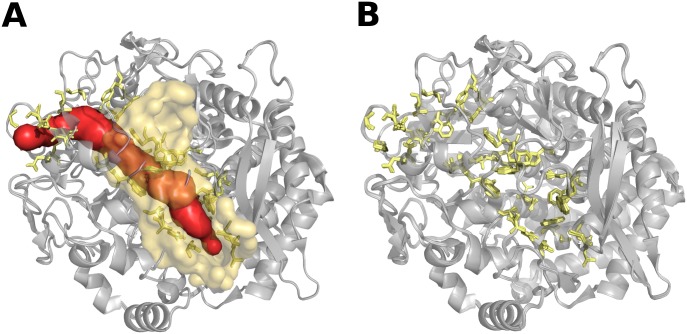
Structural superposition of the cellulose cel48F conformations from Clostridium cellulolyticum. This enzyme is a processive endo-cellulase with a large active and binding site to locate a cellulose chain which enters to the protein through a cleft located in the surface of the protein [41]. In our dataset, this protein contains 8 conformers (PDB codes: 1F9D_A, 1F9O_A, 1G9G_A, 1FAE_A, 1FBO_A, 1FBW_A, 1FCE_A, 2QNO_A) with a maximum RMSD of 0.21 Å. (A) We can see that there is almost no significant structural difference in the carbon-alpha trace between conformers in the pair of maximum RMSD (PDB codes: 1F9O_A, 1G9G_A), however, the tunnels (in red) as large as 65 Å long (predicted by MOLE) appeared in one conformer (PDB code: 1F9O_A) which contains different ligands while in another conformer this tunnel is absent. (B) The superposition of all conformers only shows slight rotations and minimal movements in lining residues (in yellow) of this main tunnel, producing the opening and closing of the tunnel. Besides our conformers comparison, molecular dynamic simulations have also confirmed the rigidity of this protein [42].

All ordered proteins have in general low conformational diversity and represents the most populated set (~60% of our dataset). This group is characterized by slight differences between conformers (mean maximum RMSD = 0.83 Å) evidencing how no large movements are required to accomplish the biological functions. We named the proteins in this set rigid. However, these proteins have larger tunnels when compared with the other sets and, importantly, the largest tunnel length variation among conformers. Apparently, backbone-independent conformational mechanisms such as the opening and closing of tunnels are required to sustain function. Tunnels are structures that allow the transit of ligands from the surface to the buried binding or active site of a protein. They can also connect buried cavities with each other and promote the exit of products [7]. As described above, residues lining these tunnels have larger RMSD than the rest of the positions in the protein. This indicates that the minimum movements for rigid proteins, are associated with the movement of functional structures such as tunnels. Interestingly, rigid proteins have on average ligands with lower molecular weight than the other two sets which also display larger cavities when compared with rigid proteins. These observations could indicate how the use of tunnels to interact with ligands could have a limitation handling larger ligands. The existence of open and closed tunnels or their size variation when conformers are compared, e.g. bottleneck dynamics [30] or conformational gating [31], may define different binding constants that could explain biological functions [32], enzyme specificity [26] and important regulatory processes such as allosterism [15]. Over the last years, allosterism without conformational change has been explained in terms of entropy effects arising from changes in the frequency and amplitude of thermal fluctuations around the mean conformation [33–35], as an alternative explanation to the classical consideration of changes in mean conformation [36,37]. The contribution of slight movements in opening and closing tunnels, pockets and cavities to this so-called entropic allosterism [38] remains to be further quantified. Several proteins where tunnels and cavities define protein function are human P450 cytochrome [39], *A*. *aeolicus* lumazine synthase [26] with more than five channels longer than 15 Å, imidazole glycerol phosphate synthase from *T*. *maritima* [40] where gating regulates the activity of two active sites, cellulose cel48F with a 65 Å long tunnel (see Fig 5A and 5B) [41,42], as well as very well studied proteins such as myoglobin and haemoglobin are all included in the rigid set derived from our study.

Disordered proteins are divided into two sets: partially disordered and malleable. Both sets have an increased conformational diversity compared to the rigid set described above. We have previously described how presence of IDRs and order-disorder transitions increases conformational diversity [21]. As protein function relates with motion, presence of IDRs could influence biological functions relying on the presence of ordered regions [43]. These two sets differ from each other in the propensity of their IDRs to appear as ordered or disordered as reflected in three main aspects. Composition of IDRs between sets is different, and possibly related with the different IDRs conformer content (see S1 Table). As previously indicated, the main reason for malleable proteins to show greater conformational diversity than partially disordered ones, is the fact that highly flexible regions (mainly loops) appear ordered and in different arrangements in malleable proteins, introducing higher RMSD values when conformers are compared.

**Fig 6:**
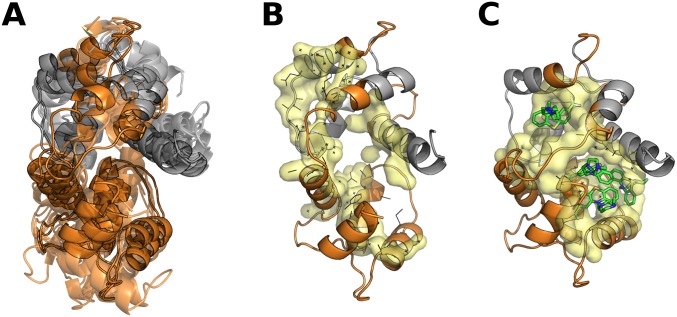
Calmodulin, a Ca2++ sensor protein. Calmodulin is a hub protein that can interact with 350 partners and it is commonly considered an ordered protein [44]. In our dataset, CaM has 79 conformers (8 with intrinsically disordered regions) with a maximum RMSD = 3.2 Å and a maximum disordered region of 34.5%. Calmodulin contains four EF-hand motifs where a pair of them form a globular domain. Each of this globular domains contains a pocket flexible enough to accommodate different target proteins through where four essential Met located in the pocket play a crucial role [45]. Additionally, each globular domain could adapt their relative orientation through flexible connectors increasing calmodulin capacity to interact with hundreds of partners. (A) We show several conformers of calmodulin superimposed (PDB codes: 1LIN_A, 1NIW_E, 3G43_A, 4DCK_B, 2FOT_A, 2X0G_B, 2BE6_A, 2O60_A, 1CDL_A, 3GP2_A). The disordered regions which are ordered in some conformers are in orange. (B-C). Whilst, in the pair of conformers with maximum RMSD, one conformation (B, PDB code: 1NIW_E) is more extent with a not well defined pocket respect to the conformation bound to trifluoperazine (C, PDB code: 1LIN_A), which is more compact and has higher cavity volume. We can show on one hand the dependence of malleable protein cavities volume with disordered regions, and on the other that the presence of these regions increase the pocket plasticity.

Partially disordered and malleable proteins have larger cavities and shorter tunnels compared with rigid proteins. This could represent different ways to interact with ligands, as cognate ligands are larger in partially disordered and malleable proteins (average number of different ligands is 4.67, 5.64 and 9.30 for rigid, partially disordered and malleable sets). However, these two protein sets have additional differences in their corresponding cavities which are also more solvent exposed and hydrophilic than the rigid set. When IDRs in any conformer are removed, cavities in malleable proteins reduce their volume to the level of rigid proteins, while partially disordered cavities remain almost unaltered. In this sense, IDRs form and define, at least partially, cavities of malleable proteins, possibly modulating the ligand interaction. This higher flexibility defining cavities, appearing disordered in a minority of conformers, is the basic structural property characterizing the set that we called malleable. A typical example is calmodulin, a hub protein that can interact with 350 partners, which does not have a high disorder level and is commonly considered an ordered protein [44,45] (Fig 6A, 6B and 6C). Other well characterized proteins with flexible binding sites include the *E*. *coli* undecaprayl-pyrophosphate synthase where flexible loops regulate the product size [46], Cyclin-dependent kinase 2 [47], Prothrombin [48] and Trypsin.

**Fig 7:**
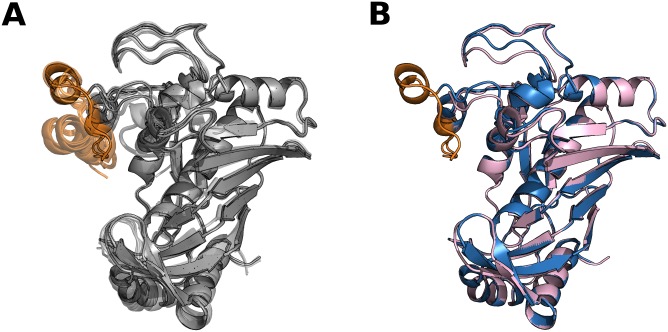
Human thymidylate synthase (TS). TS is responsible to catalyze the reductive methylation of dUMP to dTMP. This reaction is essential to maintain the nucleotide pools during cell growth. This enzyme is a valuable target for cytotoxic drugs used in cancer chemotherapy, because of its importance for DNA replication in actively dividing cells [51,52]. TS has disorder regions which plays an important role for dimmer stabilization, assembly and regulation by order to disorder transitions. The TS has 15 conformers (11 with IDR’s) in our data set with a maximum RMSD = 1.34 Å and a maximum percentage of disorder (according to our definition) of 9%. The disordered region of this protein is composed by residues from one of the inserts that presents human TS differs to bacterial TS. (A) We show all conformers of the TS superimposed (PDB codes: 1HW3_A, 1HZW_A, 1HZW_B, 1I00_A, 1I00_B, 1YPV_A, 3EDW_X, 3EHI_X, 3GH2_X, 3N5E_A, 3N5E_B, 3N5G_A, 4G2O_X, 4G6W_X, 4GD7_A). The disorder region changes from disorder to order in many conformers (residues in orange), for example, in the active conformation (PDB code = 1HZW_A) this region is ordered while in the inactive (PDB code = 1YPV_A) is disordered. (B) Pair of conformers with maximum RMSD (1YPV_A in pink, 4GD7_A in light blue). The disorder region appears partially ordered in the two conformers, the only difference is that the region in the structure 4GD7_A has eleven more ordered residues (which forms an alpha-helix) than the same region in the other conformation.

Contrary to malleable proteins, cavities are not affected by IDRs in the partially disordered set. This set shows the largest number of hinges and domains, hence we assume that IDRs could form high flexible linkers and connectors between domains [49]. This set also has the longest IDRs (see S1 Table) and about 5% of the proteins have a maximum disorder length which could involve entire domains. Due to the higher disorder content, a higher proportion of disordered conformers comprising about 70% of the IDPs in our dataset, we think that partially disordered proteins are mainly formed by very well characterized canonical IDPs which mostly agree with the current zeitgeist in IDPs [8,44,50]. In this set, we found several examples of well characterized IDPs such as the 50S ribosomal proteins L15, L11, L10 and L32e, guanine nucleotide-binding protein G and Thymidylate synthase [51,52] (see Fig 7A and 7B) are examples of order/disorder transitions related with function. Histo-blood group A transferase and Endonuclease VIII are examples of IDPs where function relates to the presence of a disorder state. An entropic chain in Serine/threonine-protein kinase PLK1 and Xanthine dehydrogenase/oxidase proteins serve just to mention some examples.

Historically, conformational diversity has been associated with large conformational changes thanks to the pioneering work by Chothia, Lesk and Gerstein [4,17]. Several examples were later compiled and extended by Gerstein in his protein motion database [5]. Where are these proteins in the sets described above? Most of the proteins show no IDRs, so we can find them in the higher RMSD tail of the rigid set. Just to measure the importance of such large motions, the 75 percentiles of rigid proteins is below 1.13 Å of RMSD. Above that number, we found 769 proteins without IDRs and with large conformational changes. Most of the changes comprise domains and/or fragments such as loops, normally as rigid bodies with hinge, shear or more complex motions [53]. The main point to stress is that this kind of motions represent about 15% of the total proteins studied here.

### Concluding remarks

The sustained advances in structural biology of the last years have been focused on the establishment of structure-function relationships to improve functional annotation of proteins displaying similar structures and/or sequences (for example [54]). This practical and useful trend hides the concept that the structure-function relationship is almost an evolutionary accident in view of the vast redundancy of similar functions sustained by different protein structures [55,56] or the contrary where similar structures achieve completely different functions(i.e. superfolds [57,58]). We have found three main conformational mechanisms expressed as structure-dynamics relationships representing different ways to achieve a vast diversity of biological functions. They are general enough to describe proteins behaviour, as early suggested by H. Frauenfelder in his search for general concepts in protein dynamics [2]. But certainly, the search for unique functional classes using low-level descriptors inside these categories appears to be a useless task.

## Methods

### Dataset generation

The CoDNaS database [59], containing a redundant collection of three dimensional structures for the same protein, was used to recruit proteins exhibiting conformational diversity. The final dataset contains 4,791 different protein chains with a total of 74,417 conformers (corresponding to 1,186,312 conformer pairs), all structures belonging to this dataset were obtained by X-Ray Diffraction at a resolution equal or less than 2.5 Å. The maximum conformational diversity for each protein is the pair of conformers with maximum C-alpha RMSD. In order to obtain a reliable and comparable estimation of conformational diversity of each protein, our dataset only contains proteins with a minimum of 5 conformers (average 15.53 conformers per protein). It is supported by the work of Best et. al which showed that is necessary to take into account a given number of structures (around 5 structures) to estimate conformational features of the native state of proteins [11] (up to 5 conformers to study backbone flexibility and around 20 to study side-chain heterogeneity). These conformers mainly differ in the presence of bound ligands (~70% on average of the conformers).

### Finding intrinsic disorder regions

We defined intrinsic disorder regions (IDRs) for each protein conformer using MobiDB [60]. If a residue has missing electron density coordinates in a structure obtained with X-ray crystallography, it assumed to be disordered [61]. To define an IDR, only five or more consecutive missing residues that were not in the amino or carboxyl terminal of the protein sequence (the first or least twenty residues) were considered. The amino acid composition was obtained from composition profile [62] using 10,000 bootstrap iterations.

### Structural characterizations

Hinges were detected with FlexProt [63], the number of hinges identified maximizes the structural superposition between conformers. The Radius of gyration (R_g_) of each conformer was calculated using the MMTSB Tool Set (available from http://blue11.bch.msu.edu/mmtsb/). Due to the dependence of R_g_ with protein size, a normalized R_g_ was calculated from the R_g_ of the ideal sphere of the same volume of the protein structure [64].

Pockets were detected with FPocket [27], using the highest scored pocket. The percentage of maximum variation between maximum pocket volumes (with/without IDRs residues) in each conformer was calculated as |max(*pV*_1_,_*IDR*_, *pV*_2_,_*IDR*_) − max(*pV*_1_, *pV*_2_)|/max(max(*pV*_1,*IDR*_, *pV*_2,*IDR*_), max(*pV*_1_, *pV*_2_)), where *pV*_*i*_ is the pocket volume (with/without IDRs residues) on the corresponding conformer. MOLE 2.0 [65] was used for tunnel identification and characterization using the following parameters. Probe Radius and Origin Radius 3 Å, Interior Threshold 1.25 Å and default values for all the others. We parsed the XML file output to identify tunnel length, number of tunnels, residues lining each tunnel and so on. The proportion of variation between largest tunnels in each conformer was calculated as |*L*_1_ − *L*_2_ |/max(*L*_1_,*L*_2_), where L_i_ is the length of the largest tunnel on the corresponding conformer in the maximum pair. The protein volume was estimated by 3vee [66] using a probe radius of 0.5 Å and a grid resolution of 1.5 Å. Residue interactions networks (RINs) were built with RING [29].

## Supporting information

S1 Table Summary of different descriptors reported on the ‘*Results*’ section. The values correspond to the mean (with the confidence interval at 95%), standard deviation and median in each compared distribution. See Methods section for details about descriptors.
(XLSX)

S2 Table Composition of *apo*/*holo* subset. The values correspond to the mean (confidence interval at 95%), standard deviation and median in each compared distribution.
(XLSX)

S1 Fig Maximum RMSD distributions at different crystallization temperatures. (A) Representative maximum pair of conformers for each protein obtained at room-temperature (above 200 K) and Cryogenic temperature (100 K). (B) The three sets in a subset of proteins in which the conformers of the maximum pair of RMSD has been crystallised at cryogenic temperature (100 K). This subset represents the 67% of the total proteins in our dataset. (C) Idem (A) which pairs obtained at room-temperature. This subset represents the 9% of the total proteins in our dataset.
(TIF)

S2 Fig RMSD vs. mean number of crystal contacts, calculated for pairs of conformers with maximum conformational diversity for each monomeric protein in our dataset. We have used a subset of monomeric proteins from our dataset, with only one protein chain in the asymmetric unit (392 pairs of conformers) in order to remove hetero biological complexes. For each conformer in the maximum pair of RMSD, we estimated the average number of crystallographic contacts (at 4.5 Å of distance) between each atom of the protein chain in the asymmetric unit and the neighbour molecules in other unit cells. We obtained a negligible Spearman’s correlation coefficient of 0.048.
(TIF)

S3 Fig Amino acid composition of IDRs. Amino acid composition of IDRs presents in all conformers of malleable (light blue) and partially disordered (yellow) proteins relative to PDB Select 25. DisProt is used as a reference of experimental protein disorder.
(TIF)

S4 Fig (A–C) Distributions of RMSD values for maximum pairs of conformational diversity in each set discriminated by secondary structure. (TIF)

S5 Fig Number of hinges distribution in maximum pairs of conformational diversity in each set. A single bar represents the relative frequency of a given number of hinges.
(TIF)

S6 Fig Mean normalized Radius of gyration distributions between conformers in maximum pair of conformational diversity. Rigid proteins show an average significantly lower than partially disordered proteins (Wilcoxon rank-sum test P << 0.001).
(TIF)

S7 Fig Maximum conformational diversity distribution of the *apo*/*holo* subset. (TIF)

S1 Dataset Datasets used in this study. (TAR)
