# Supplementary figures and images for: Conformational diversity analysis reveals three functional mechanisms in proteins
Source: PLoS Comput Biol. 2017 Feb 13;13(2):e1005398. doi: 10.1371/journal.pcbi.1005398 (PMC5330503; doi:10.1371/journal.pcbi.1005398)

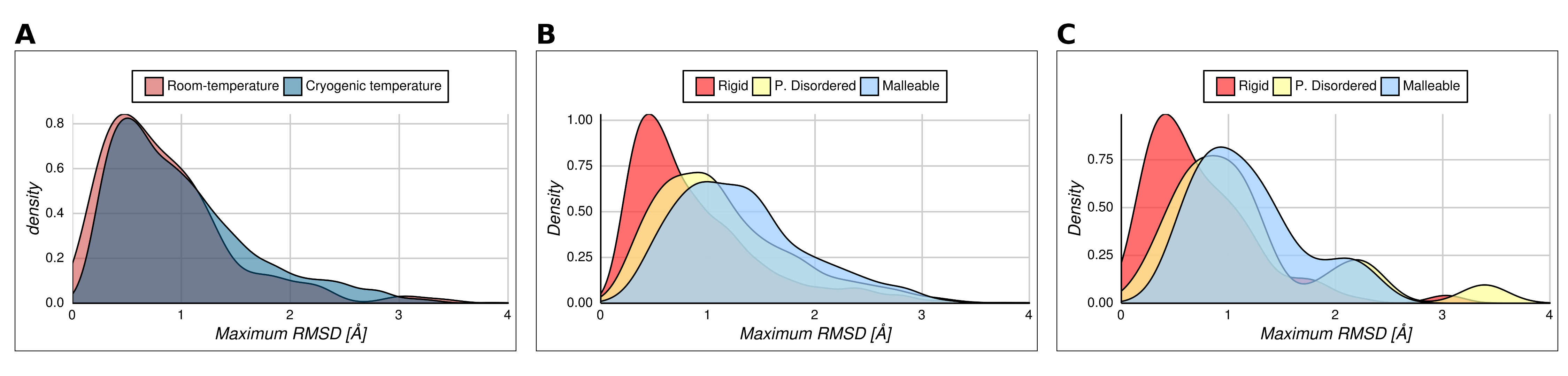

Supplement: S1 Fig — (A) Representative maximum pair of conformers for each protein obtained at room-temperature (above 200 K) and Cryogenic temperature (100 K). (B) The three sets in a subset of proteins in which the conformers of the maximum pair of RMSD has been crystallised at cryogenic temperature (100 K). This subset represents the 67% of the total proteins in our dataset. (C) Idem (A) which pairs obtained at room-temperature. This subset represents the 9% of the total proteins in our dataset. (TIF) [file pcbi.1005398.s003.tif]

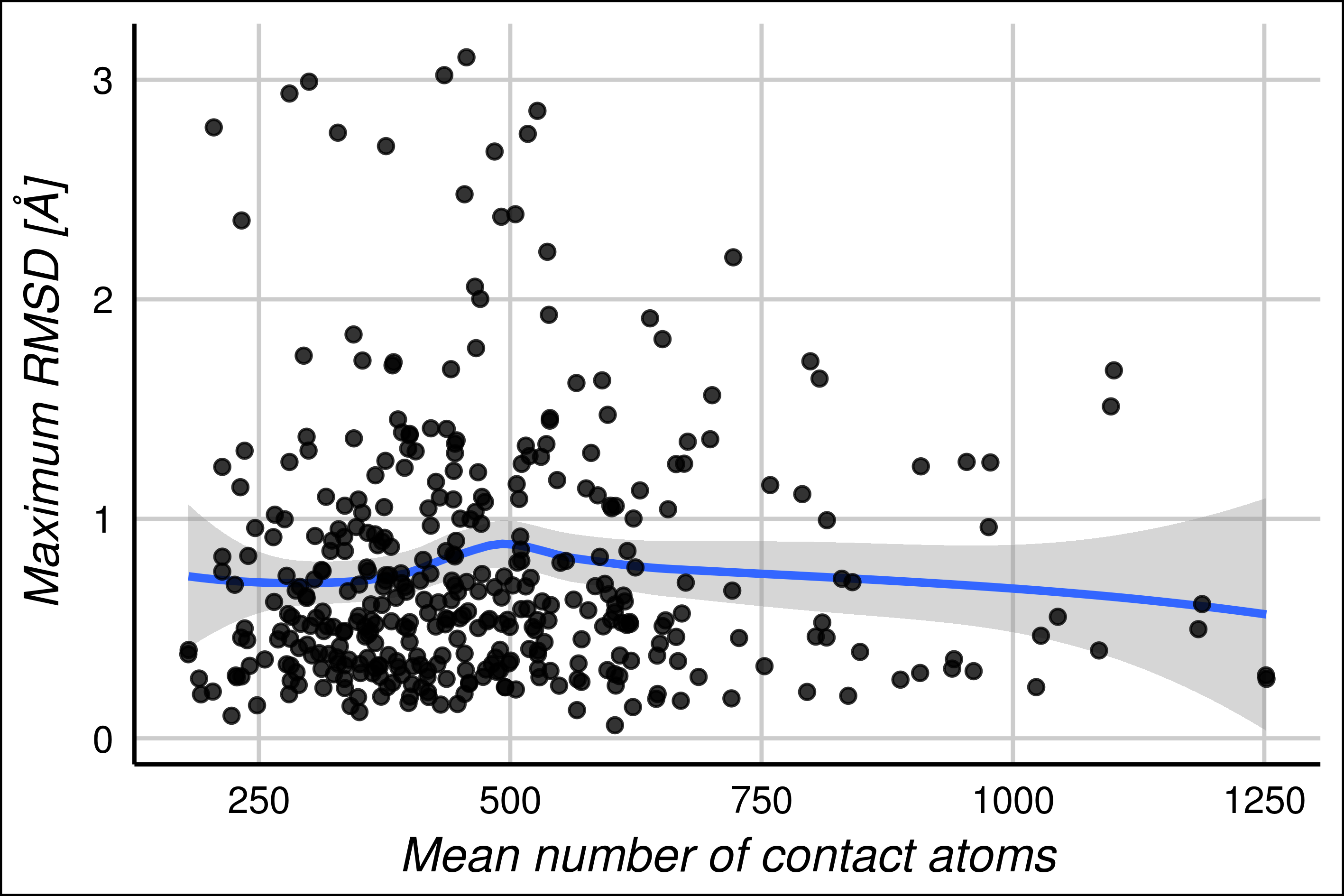

Supplement: S2 Fig — We have used a subset of monomeric proteins from our dataset, with only one protein chain in the asymmetric unit (392 pairs of conformers) in order to remove hetero biological complexes. For each conformer in the maximum pair of RMSD, we estimated the average number of crystallographic contacts (at 4.5 Å of distance) between each atom of the protein chain in the asymmetric unit and the neighbour molecules in other unit cells. We obtained a negligible Spearman’s correlation coefficient of 0.048. (TIF) [file pcbi.1005398.s004.tif]

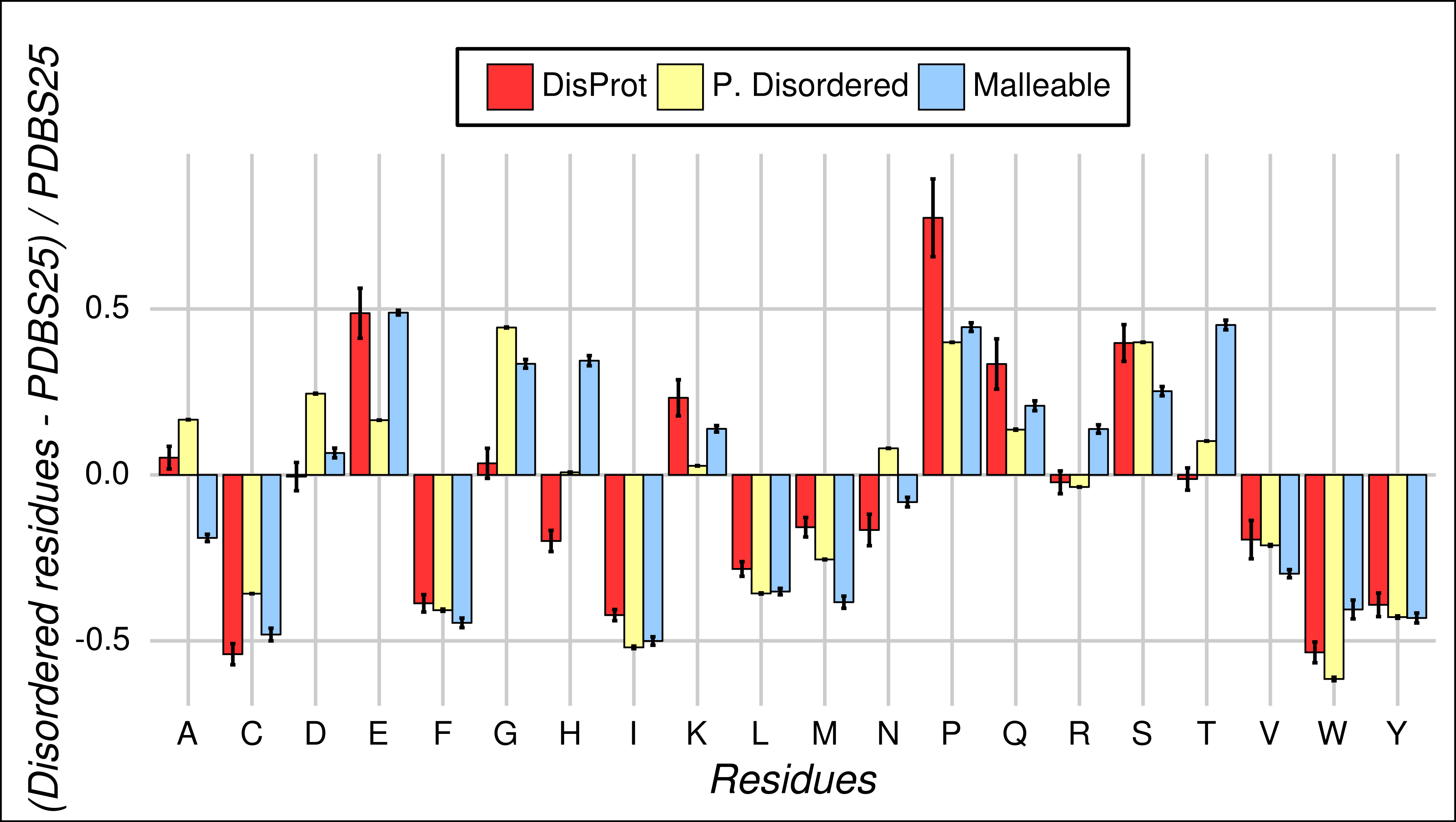

Supplement: S3 Fig — Amino acid composition of IDRs presents in all conformers of malleable (light blue) and partially disordered (yellow) proteins relative to PDB Select 25. DisProt is used as a reference of experimental protein disorder. (TIF) [file pcbi.1005398.s005.tif]

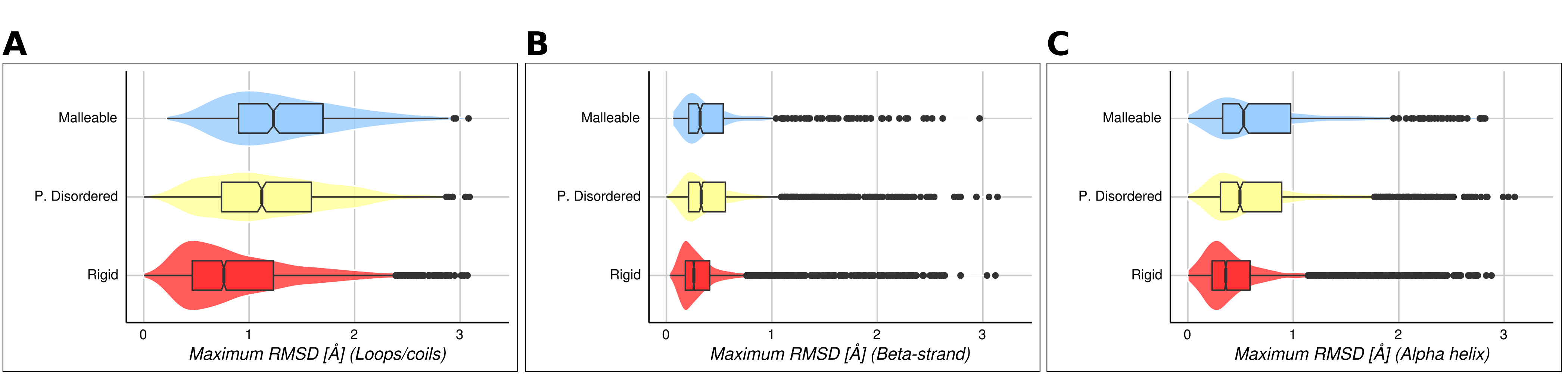

Supplement: S4 Fig — (TIF) [file pcbi.1005398.s006.tif]

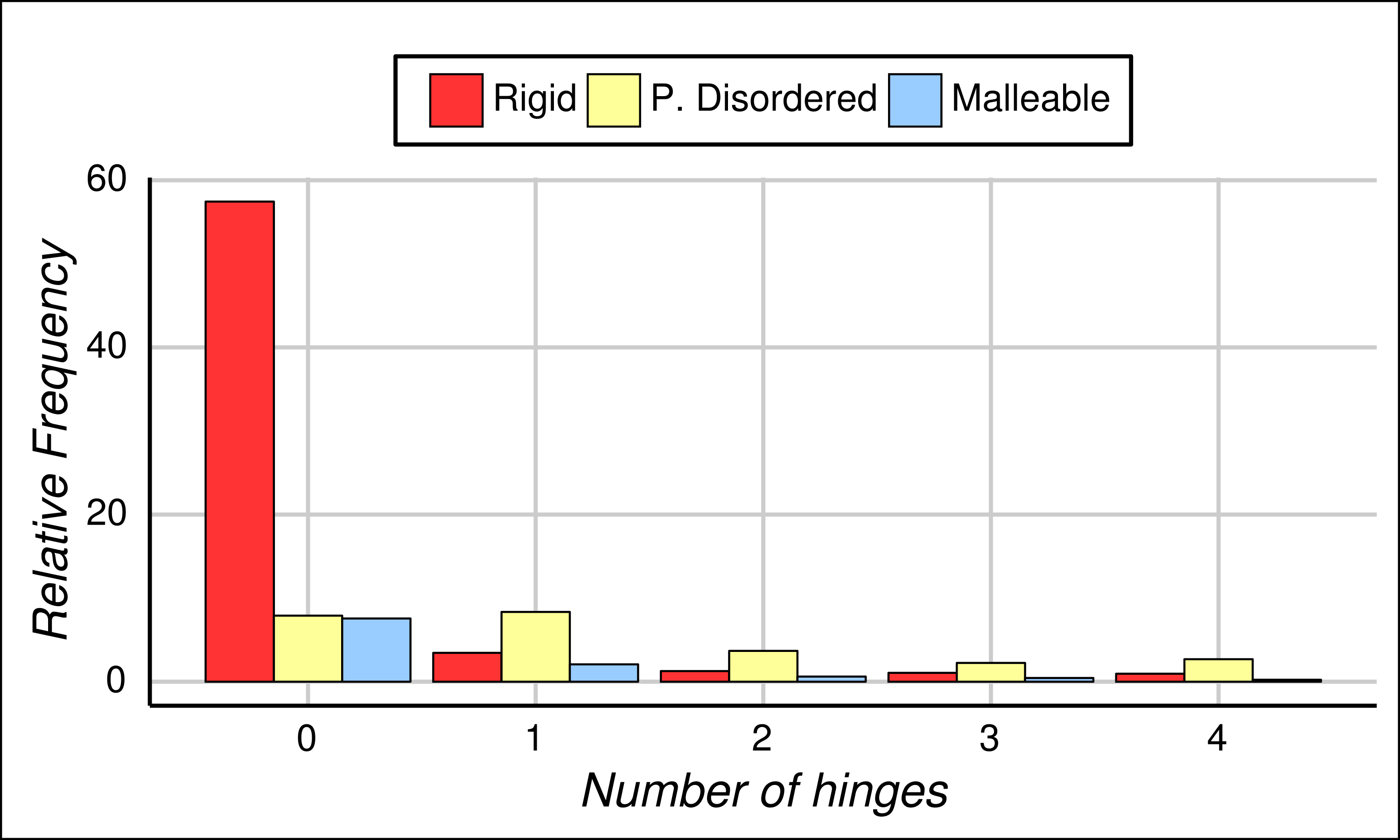

Supplement: S5 Fig — A single bar represents the relative frequency of a given number of hinges. (TIF) [file pcbi.1005398.s007.tif]

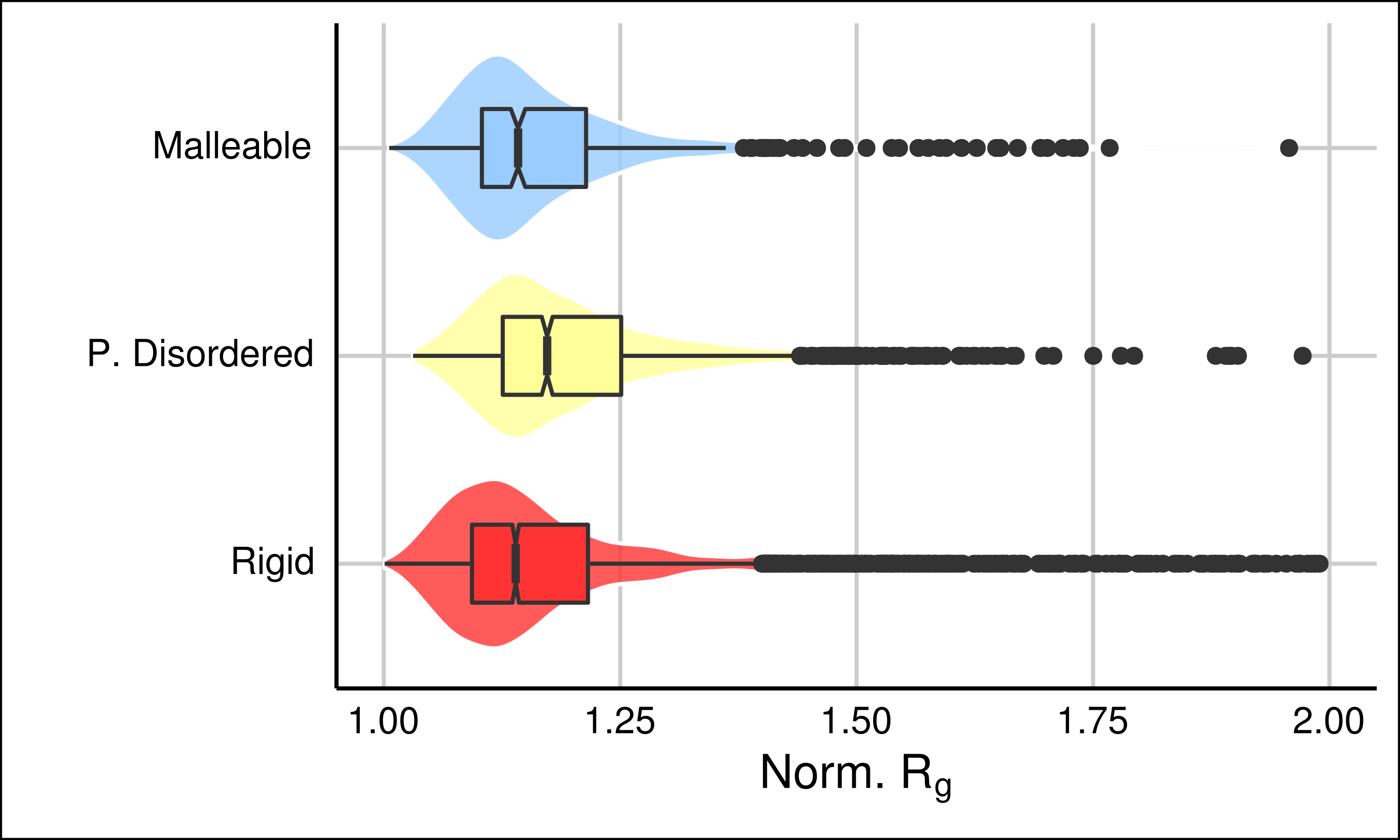

Supplement: S6 Fig — Rigid proteins show an average significantly lower than partially disordered proteins (Wilcoxon rank-sum test P << 0.001). (TIF) [file pcbi.1005398.s008.tif]

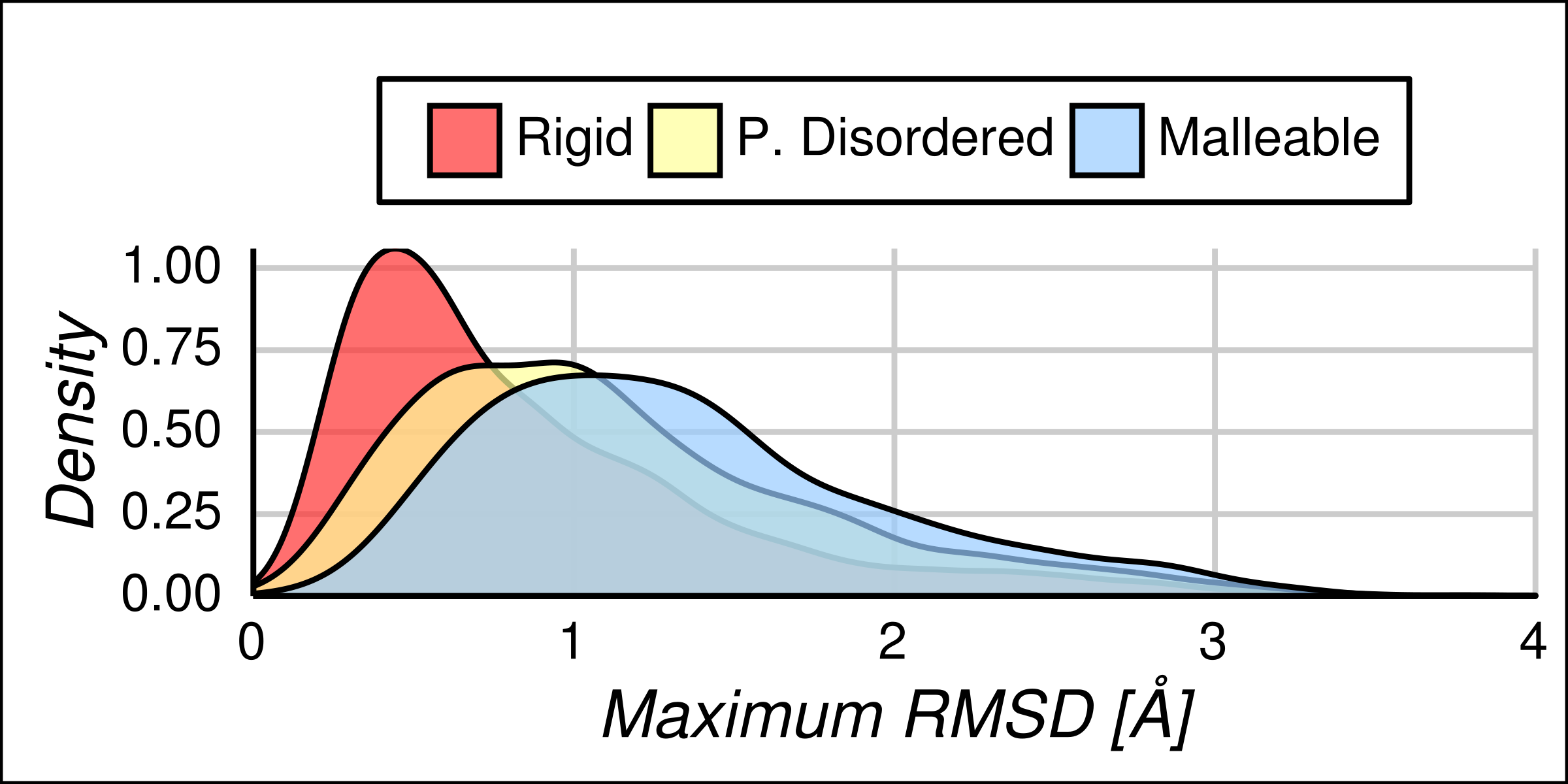

Supplement: S7 Fig — (TIF) [file pcbi.1005398.s009.tif]
